# The substantial hospitalization burden of influenza in central China: surveillance for severe, acute respiratory infection, and influenza viruses, 2010–2012

**DOI:** 10.1111/irv.12205

**Published:** 2013-11-10

**Authors:** Hongjie Yu, Jigui Huang, Yang Huai, Xuhua Guan, John Klena, Shali Liu, Youxing Peng, Hui Yang, Jun Luo, Jiandong Zheng, Maoyi Chen, Zhibin Peng, Nijuan Xiang, Xixiang Huo, Lin Xiao, Hui Jiang, Hui Chen, Yuzhi Zhang, Xuesen Xing, Zhen Xu, Zijian Feng, Faxian Zhan, Weizhong Yang, Timothy M Uyeki, Yu Wang, Jay K Varma

**Affiliations:** a Divison of Infectious Disease, Chinese Center for Disease Control and PreventionBeijing, China; b Jingzhou Center for Disease Control and PreventionJingzhou, China; c China-US Collaborative Program on Emerging and Re-emerging Infection Disease, US Centers for Disease Control and PreventionBeijing, China; d Hubei Provincial Centre for Disease Control and PreventionWuhan, China; e Jingzhou Central HospitalJingzhou, China; f Jingzhou First People's HospitalJingzhou, China; g Jingzhou Second People's HospitalJingzhou, China; h Jingzhou Maternal and Children's HospitalJingzhou, China; i Public Health Emergency Center, Chinese Center for Disease Control and PreventionBeijing, China; j Chinese Center for Disease Control and PreventionBeijing, China; k Influenza Division, National Center for Immunization and Respiratory Diseases, Centers for Disease Control and PreventionAtlanta, GA, USA; l Global Disease Detection Program, Division of Global Disease Detection and Response, Center for Global Health, Centers for Disease Control and PreventionAtlanta, GA, USA

**Keywords:** China, disease burden, influenza, seasonality

## Abstract

**Background:**

Published data on influenza in severe acute respiratory infection (SARI) patients are limited. We conducted SARI surveillance in central China and estimated hospitalization rates of SARI attributable to influenza by viral type/subtype.

**Methods:**

Surveillance was conducted at four hospitals in Jingzhou, China from 2010 to 2012. We enrolled hospitalized patients who had temperature ≥37·3°C and at least one of: cough, sore throat, tachypnea, difficulty breathing, abnormal breath sounds on auscultation, sputum production, hemoptysis, chest pain, or chest radiograph consistent with pneumonia. A nasopharyngeal swab was collected from each case-patient within 24 hours of admission for influenza testing by real-time reverse transcription PCR.

**Results:**

Of 17 172 SARI patients enrolled, 90% were aged <15 years. The median duration of hospitalization was 5 days. Of 16 208 (94%) SARI cases tested, 2057 (13%) had confirmed influenza, including 1427 (69%) aged <5 years. Multiple peaks of influenza occurred during summer, winter, and spring months. Influenza was associated with an estimated 115 and 142 SARI hospitalizations per 100 000 during 2010–2011 and 2011–2012 [including A(H3N2): 55 and 44 SARI hospitalizations per 100 000; pandemic A(H1N1): 33 SARI hospitalizations per 100 000 during 2010–2011; influenza B: 26 and 98 hospitalizations per 100 000], with the highest rate among children aged 6–11 months (3603 and 3805 hospitalizations per 100 000 during 2010–2011 and 2011–2012, respectively).

**Conclusions:**

In central China, influenza A and B caused a substantial number of hospitalizations during multiple periods each year. Our findings strongly suggest that young children should be the highest priority group for annual influenza vaccination in China.

## Background

Historically, influenza surveillance has focused on outpatient visits for influenza-like illness (ILI; defined as temperature ≥38°C with either cough or sore throat and no alternative diagnosis) and, among those with ILI, the proportion with laboratory-confirmed influenza, stratified by viral type/subtype.^1^ Influenza surveillance in outpatients has informed current understanding about the seasonality and characteristics of influenza viruses, data that have helped determine the timing and composition of annual influenza vaccination.^2^ Recently, the World Health Organization (WHO) has recommended that influenza surveillance also include sentinel surveillance for severe acute respiratory infection (SARI), which is often defined as ILI plus one additional symptom or sign of severe illness in a hospitalized patient.^3^ The rationale is that severe outcomes of influenza have the greatest public health and economic impact and that identifying risk factors for severe illness informs vaccine policy.

The burden of influenza, specifically hospitalization rates attributable to laboratory-confirmed influenza, is well documented in industrialized countries^4^–^8^ but less so in developing countries and in subtropical regions.^9^–^13^ China is a lower middle-income country with the largest population (1·3 billion) in the world. The burden of laboratory-confirmed influenza among a defined general Chinese population has not been thoroughly studied. Although seasonal influenza vaccine use has increased dramatically, the vast majority of the Chinese population is not vaccinated annually.^14^

During 2010, in the post-2009 H1N1 pandemic period, we initiated active surveillance for SARI in a central Chinese city. We analyzed data from this system during a 24-month period to characterize the epidemiology and estimate hospitalization rates attributable to laboratory-confirmed influenza.

## Methods

### Setting

Surveillance was conducted at four large hospitals, including three general hospitals and one pediatric hospital, located within two districts of Jingzhou City, Hubei Province, China. In 2010, the total population residing within these two districts was 1 154 086, of which 122, 301 (11%) were children aged <15 years, and 39 729 (3%) were aged <5 years.^15^ The mean annual temperature in this humid subtropical city is 16·5°C, and the average rainfall is 1100–1300 mm.^16^

Before initiating surveillance, we reviewed medical records to obtain International Classification of Diseases 10th Revision (ICD-10) discharge diagnoses for all healthcare facilities that provide inpatient services in these two districts. The four hospitals in our surveillance system consistently accounted for the majority of hospitalizations in the two districts whether for all discharge diagnoses (median 65%, range: 62–73%) between 2006 and 2010 or for age-specific influenza-associated primary discharge diagnoses (median 70%, range: 69–71%, ICD-10 codes: H66, J00-J99, P23.9, R50.9, I27.9) between 2006 and 2008 (Table S1).

### Patient enrollment

All patients admitted to a surveillance hospital were screened by nurses and physicians for SARI. A patient was defined as having SARI if they had an elevated temperature (rectal or axillary temperature ≥37·3°C) and at least one sign or symptom of acute respiratory illness, including cough, sore throat, tachypnea, difficulty breathing, abnormal breath sounds on auscultation, sputum production, hemoptysis, chest pain, or chest radiograph consistent with pneumonia. Patients were considered eligible if the SARI case definition was met within 24 hours of hospital admission. We excluded newborns born in a surveillance hospital and not yet discharged after birth.

This project was approved by the ethical review committees at the Chinese Center for Disease Control and Prevention (China CDC, Beijing, China) and the Centers for Disease Control and Prevention (US CDC, Atlanta, United States). In response to pH1N1, China's Ministry of Health implemented new national influenza surveillance guidelines in October 2009; for this project, therefore, study participation only required patients or their parent/guardian to provide brief verbal consent.

### Patient data collection

After hospital admission, physicians obtained verbal consent from eligible patients or their parent/guardians and then completed a standardized case report form. At hospital discharge, physicians were required to update the form to include data about clinical course in the hospital, including treatments received, complications, and outcome. Public health staff telephoned patient homes up to 3 months after discharge to document whether patients were alive 30 days after discharge.

### Specimen collection and testing

Nurses collected nasopharyngeal (NP) swabs from SARI case-patients within 24 hours of admission following standardized procedures. Swabs were placed immediately in viral transport medium (VTM) and stored at 4°C at the local hospital. Specimens were transferred three times per week, most within 48 hours of collection, to the Jingzhou Center for Disease Control and Prevention (Jingzhou CDC). At Jingzhou CDC, specimens in VTM were stored at −70°C until testing by real-time reverse transcription PCR (rRT-PCR). Viral RNA was extracted from 200 μl of VTM using an RNeasy Mini kit (Qiagen, Dusseldorf, Germany) per the manufacturer's protocols. RNA from each sample was tested for specific primers and probes that target influenza A, influenza B, pH1N1, seasonal A (H1N1), and seasonal A (H3N2), following the US CDC's protocol.^17^ These assays were performed in biosafety level 2 facilities of Jingzhou CDC, which undergoes quality control assessment by the National Influenza Center at the China CDC.

### Data analysis

Hospital and Jingzhou CDC staff entered data into an electronic database that was transmitted monthly to the China CDC. After a three-month pilot period that began in January 2010, we collected data continuously. We analyzed data that were collected from April 5, 2010–April 8, 2012 with spss (v13.0; SPSS, Chicago, IL, USA). We defined a patient with laboratory-confirmed influenza as any SARI case-patient with an NP swab that tested positive for influenza virus by rRT-PCR.

We estimated hospitalization rates for SARI, stratified by age group, by adjusting for the size of the resident population in the two districts and the age-specific proportion of all influenza-associated hospitalized patients at the four surveillance hospitals. We estimated hospitalization rates of SARI attributable to laboratory-confirmed influenza, adjusted for the proportion of SARI patients that had NP swabs collected for influenza testing. We excluded data for non-residents from the numerators and denominators to estimate hospitalization rates. We adjusted the proportion for all hospitalized patients, instead of the proportion of influenza-associated hospitalized patients, evaluated in the four surveillance hospitals.

## Results

### Characteristics of SARI patients

**Figure 1:**
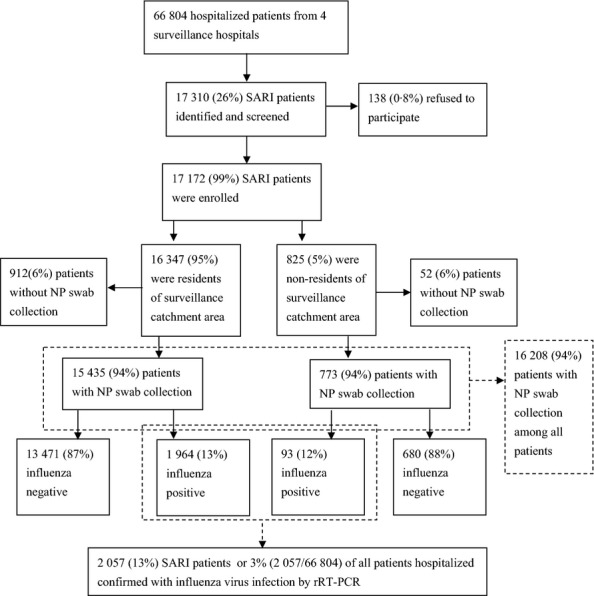
Enrollment of 17 172 severe acute respiratory infection (SARI) patients and 2057 SARI patients confirmed with influenza virus infection during 24 months of surveillance (from April 5, 2010 to April 8, 2012) in Jingzhou, China.

From April 5, 2010–April 8, 2012, 66 804 patients were hospitalized in the four surveillance hospitals, and 17 172 (26%) patients with SARI were enrolled (Figure 1). Among patients with SARI, 95% were residents of the surveillance districts. The vast majority (90%) of SARI cases were in children aged <15 years, with a median age of 2·2 years [interquartile range (IQR), 1–4·4 years]. Few SARI case-patients (7%) had a chronic medical condition (Table 1). A temperature >38°C was documented in 60% of SARI case-patients at the time of physical examination; of note, the case report form only recorded the patient's temperature at the time they were examined on the inpatient ward, not their temperature at presentation to the emergency department or outpatient clinic. Cough was the most common symptom (60%).

**Table 1:** Characteristics of hospitalized severe, acute respiratory infection (SARI) patients and laboratory-confirmed influenza patients in four surveillance hospitals in Jingzhou, China, April 5, 2010 to April 8, 2012

Characteristics	All SARI patients (%) [*n* = 17 172]*	SARI patients with confirmed influenza (%) [*n* = 2057]*	SARI patients without confirmed influenza (%) [*n* = 14 151]*	*P* value**
Male sex	5867 (34)	1195 (58)	8097 (57)	0·453
Age, median [interquartile range (IQR), years]	2·2 (1·0–4·4)	2·9 (1·2–6·0)	2 (1·0–4·1)	
Age group				
<6 months	1318 (8)	156 (8)	1099 (8)	
6–11 months	3121 (18)	263 (13)	2684 (19)	
12–23 months	3596 (21)	339 (17)	3042 (22)	
2–4 years	5318 (31)	669 (33)	4361 (31)	
5–9 years	1710 (10)	300 (15)	1300 (9)	
10–14 years	319 (2)	49 (2)	242 (2)	
15–49 years	617 (4)	93 (5)	495 (4)	
50–64 years	454 (3)	77 (4)	360 (3)	
≥65 years	719 (4)	111 (5)	568 (4)	
Underlying chronic medical conditions				
At least one***	1200 (7)	184 (9)	951 (7)	<0·001
Chronic obstructive pulmonary disease†	148 (0·9)	27 (1)	111 (0·8)	0·015
Asthma	101 (0·6)	20 (1)	77 (0·5)	0·019
Cardiovascular disease	116 (0·7)	20 (1)	88 (0·6)	0·068
Diabetes mellitus	86 (0·5)	12 (0·6)	71 (0·5)	0·628
Children <2 years††				
Low birth weight†††	82/8035 (1)	13/758 (2)	67/6825 (1)	0·061
Premature birth‡	117/8035 (2)	17/758 (2)	97/6825 (1)	0·078
Clinical history and physical examination				
Temperature ≥38°C	10 279 (60)	1319 (64)	8393 (59)	0·001‡‡
Abnormal breath sounds on auscultation	5610 (33)	715 (35)	4611 (33)	0·050
Cough	10 240 (60)	1408 (68)	8284 (59)	<0·001
Sore throat	4444 (26)	461 (22)	3794 (27)	<0·001
Runny nose	2279 (13)	341 (17)	1811 (13)	<0·001
Sputum production	2188 (13)	307 (15)	1750 (12)	0·001
Difficulty breathing	892 (5)	93 (5)	740 (5)	0·174
Documented tachypnea‡‡‡	255 (2)	36 (2)	207 (2)	0·316
Chest pain	143 (0·8)	17 (0·8)	125 (0·9)	0·796
Hemoptysis	77 (0·4)	12 (0·6)	60 (0·4)	0·310
Vomiting	913 (5)	74 (4)	767 (5)	<0·001
Diarrhea	635 (4)	30 (2)	564 (4)	<0·001
Abdominal pain	254 (2)	29 (1)	209 (2)	0·813
Clinical pneumonia	4885 (28)	576 (28)	4062 (29)	0·510
Chest X-ray performed	8486 (49)	1066 (52)	6903 (49)	0·010
The presence of radiographic diagnosis of pneumonia	3814/8486 (45)	430/1066 (40)	3180/6903 (46)	<0·001
Clinical course, median (IQR), days				
From illness onset to hospital admission	2 (0–3)	2 (0–3)	2 (0–3)	0·343
Length of stay in hospital	5 (4–7)	5 (4–7)	5 (4–7)	0·469
Admission to ICU§	154 (0·9)	22 (1)	126 (0·9)	0·425
Death§§	62 (0·4)	8 (0·4)	51 (0·4)	0·841

* Data are presented as no. (%) of patients unless otherwise indicated. Denominators for testing of fewer cases than full group are indicated. Percentages may not total 100 because of rounding. There are a certain proportion of case-patients with no influenza testing results, so patients with and without confirmed influenza are not total SARI patients.

** The *P*-values are comparisons between ‘SARI patients with confirmed influenza’ and ‘SARI patients without confirmed influenza’.

*** Atleast one underlying medical condition defined as: an inpatient stays with admission diagnosis in any of the following disease, symptoms or signs: chronic obstructive pulmonary disease, asthma, cardiovascular disease, diabetes chronic metabolic disease, or renal dysfunction.

† Chronic obstructive pulmonary disease defined as: a lung disease characterized by chronic obstruction of lung airflow that interferes with normal breathing and is not fully reversible.

†† Denominator is patients <2 years old with complete information for this variable.

††† Low birth weight defined as a weight of <2500 g (up to and including 2499 g), irrespective of gestational age.

‡ Premature birth defined as a birth that takes place before 37 weeks of gestation have passed.

‡‡ Compare with patients with normal temperature.

‡‡‡ Documented tachypnea defined as: elevated respiratory rate in different age groups: (i) Age < 2 months: >60 breaths/minute; (ii) Age 2–11 months: >50 breaths/minute; (iii) Age 12 months to <5 years: >40 breaths/minute; and (iv) Age ≥5 years: >25 breaths/minute.

§ ICU denotes intensive care unit.

§§ During hospitalization or up to 30 days after hospital discharge.

Of 8486 (49%) of 17 172 SARI patients that had a chest X-ray performed, 3814(45%) were reported to have radiographic evidence of pneumonia. Of 4329 (89%) of 4885 SARI patients clinically diagnosed with pneumonia that had a chest X-ray performed, 3814 (88%) were reported to have radiographic evidence of pneumonia. The median duration of hospitalization for SARI patients was 5 days (IQR, 4–7), and 154 (0·9%) patients were admitted to an intensive care unit. Sixty-two (0·4%) patients died either in the hospital or within 30 days after discharge (Table 1).

### Characteristics of patients with laboratory-confirmed influenza

Of SARI cases, 16 208 (94%) had an NP swab specimen collected, and 2057 (13%) tested positive for influenza viruses by rRT-PCR, of which 1776 (86%) were children aged <15 years and 1427 (69%) were aged <5 years. Characteristics of SARI patients who had NP swabs collected were similar to those who did not have NP swabs collected (Table S2). Influenza viruses detected included A (H3N2) (785, 38%), pH1N1 (274, 13%), and influenza B (998, 49%). Severe acute respiratory infection patients with confirmed influenza had a significantly higher median age than those without confirmed influenza (2·9 years versus 2), and more often had at least one chronic medical condition (9% versus 7%) including chronic obstructive pulmonary disease and asthma (*P* < 0·01 for both comparisons). Compared with SARI patients without confirmed influenza, those with confirmed influenza more frequently had cough, runny nose, and sputum production, but less often had sore throat, vomiting, and diarrhea (Table 1). Eight deaths occurred among SARI case-patients with confirmed influenza including three that died during hospitalization and five that died within 30 days after discharge.

### Temporal trends

**Figure 2:**
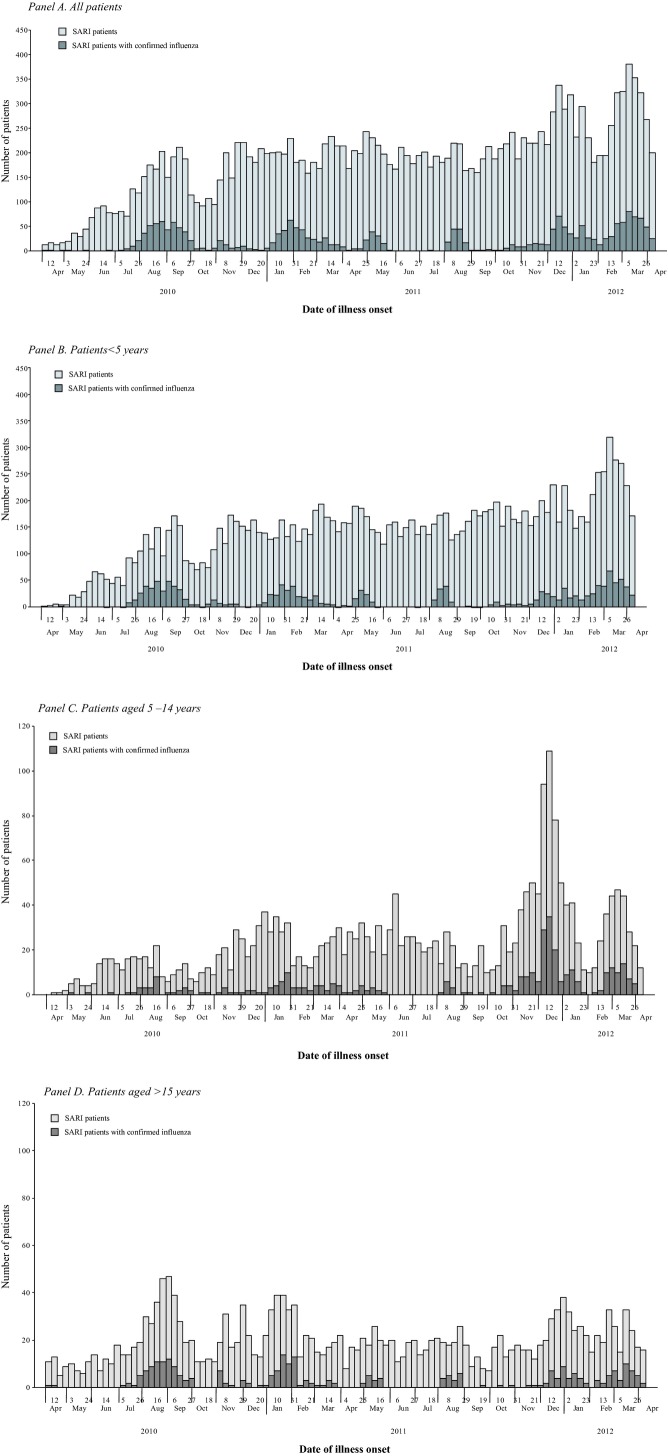
Hospitalized severe acute respiratory infection patients (*N* = 17 172) and patients confirmed with influenza viral infection (*n* = 2057) by date of illness onset, Jingzhou, China, April 5, 2010 to April 8, 2012. (Panel A) All patients. (Panel B) Patients <5 years. (Panel C) Patients aged 5–14 years. (Panel D) Patients aged >15 years.

**Figure 3:**
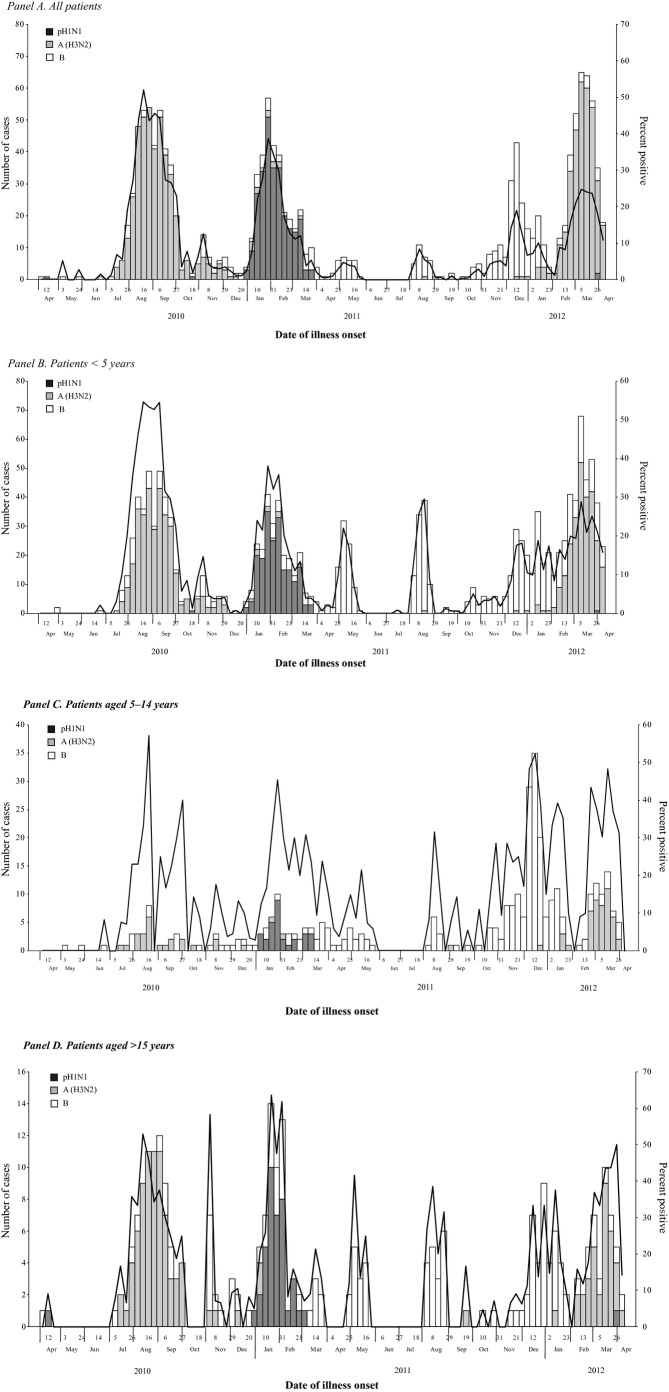
Number and percent of influenza positive by type/subtype and by week of illness onset among hospitalized severe acute respiratory infection patients (*N* = 17 172), Jingzhou, China, April 5, 2010 to April 8, 2012. (Panel A) All patients. (Panel B) Patients <5 years. (Panel C) Patients aged 5–14 years. (Panel D) Patients aged >15 years.

Over the 24-month period, there was no clear seasonal peak for SARI patients, though there were relative increases in influenza activity during summer (August and September), winter (December to February), and spring (March to May) months (Figure 2, Panel A). This pattern was consistent across different age groups (Figure 2, Panel B–D). Influenza viruses associated with SARI were predominantly A (H3N2) during 2010 summer months, pH1N1 during 2010–2011 winter months, B during 2011 spring and summer and 2011–2012 winter months, and A (H3N2) during 2012 spring months (Figure 3, Panel A). This pattern was also consistent across different age groups (Figure 3, Panel B–D).

### Hospitalization rates associated with influenza

**Figure 4:**
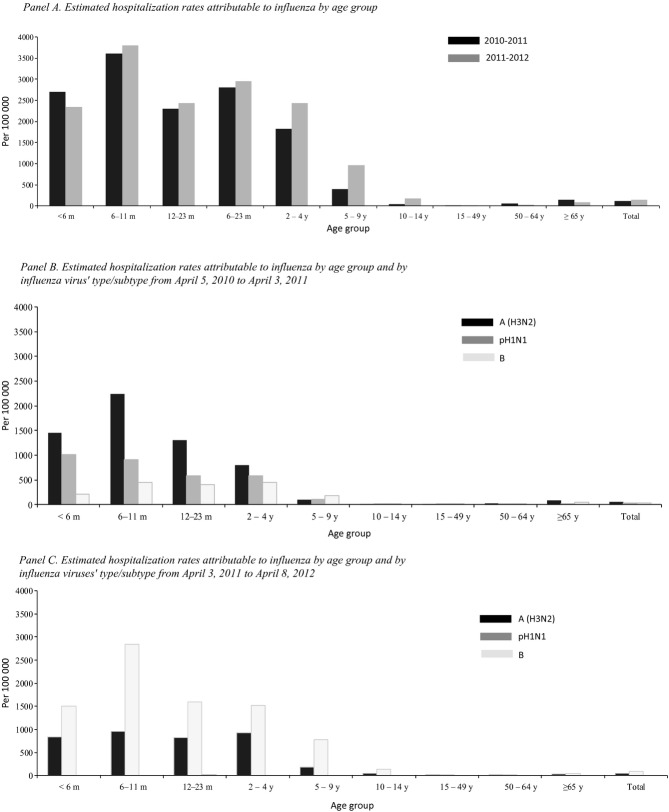
Estimated hospitalization rates attributable to influenza by age group and by type/subtype in Jingzhou, China, April 5, 2010 to April 8, 2012. (Panel A) Estimated hospitalization rates attributable to influenza by age group. (Panel B) Estimated hospitalization rates attributable to influenza by age group and by influenza viruses' type/subtype from April 5, 2010 to April 3, 2011. (Panel C) Estimated hospitalization rates attributable to influenza by age group and by influenza viruses' type/subtype from April 3, 2011 to April 8, 2012.

Of 2057 SARI patients with laboratory-confirmed influenza, 1964 (95%) were residents of the surveillance districts. Overall, influenza was associated with an estimated 115 SARI hospitalizations per 100 000 during 2010–2011 and 142 per 100 000 during 2011–2012 among all ages, with the highest rates among children aged 6–11 months (3603 and 3805 hospitalizations per 100 000 during 2010–2011 and 2011–2012, respectively). Influenza-associated SARI mostly affected children aged <5 years (2021 hospitalizations per 100 000 during 2010–2011and 2349 per 100 000 during 2011–2012), and rates declined with increasing age until age 50 years. For adults aged ≥65 years, influenza-associated SARI rates were 141 and 89 hospitalizations per 100 000 during 2010–2011 and 2011–2012, respectively. Influenza-associated SARI hospitalization rates varied slightly during the two seasons, but the age distribution was similar (Figure 4, Panel A and Table S3).

### Hospitalization rates associated with influenza by type/subtype

Among all ages, A (H3N2) virus was associated with an estimated 55 SARI and 44 hospitalizations per 100 000 during 2010–2011 and 2011–2012, respectively; pH1N1 virus was associated with 33 SARI hospitalizations per 100 000 during 2010–2011, but only two case-patients during 2011–2012; and influenza B virus was associated with 26 and 98 SARI hospitalizations per 100 000 during 2010–2011 and 2011–2012, respectively. The highest SARI hospitalization rates associated with H3N2 and B in the two seasons were among children aged 6–11 months. For pH1N1, the highest SARI hospitalization rate was among children aged <6 months and decreased as age increased, during 2010–2011 (Figure 4, Panel B and C).

## Discussion

Our study is the first in China to estimate hospitalization rates attributable to influenza by viral type/subtype among a well-defined population. We found that a substantial number of patients are hospitalized annually with influenza, especially children aged <5 years. Hospitalization rates varied by influenza type/subtype, with much higher rates associated with influenza B than A(H3N2) during 2011–2012. Influenza hospitalizations varied throughout the year with peaks in summer, winter, and spring months, in contrast with the distinct winter-only peak observed in Northern China.^18^

Among all cause hospitalizations in young children aged <5 years in the surveillance hospitals, we estimate that 35% were for SARI during the two-year-study period, including 4% with laboratory-confirmed influenza. Influenza-associated SARI hospitalization rates were high for children aged <5 years in each of the two seasons, but were sharply lower for those aged ≥5 years. Similarly high hospitalization rates have also been documented for seasonal influenza in subtropical regions such as Hong Kong SAR, Bangladesh and Mexico, with estimated rates varying from 380 to 670 per 100 000 population among children aged <5 years.^11^–^13^ Studies conducted in the temperate USA, which used different methodologies and denominators, reported estimated hospitalization rates for laboratory-confirmed influenza of 8·6 per 10 000 children, 3–4 per 1000 children aged <2 years/year and 5–151 per 100 000 per person-months for children aged <5 years.^5^,^6^,^8^ Our findings are consistent with a recent global systematic review, which estimated 3500 influenza-associated acute lower respiratory infections (ALRI) occurred in developing countries and 200 influenza-associated severe ALRI cases in the WHO Western Pacific region per 100 000 for children younger than 5 years in 2008, respectively.^19^ Studies in temperate countries, such as USA, have reported the highest influenza-associated hospitalization rates in infants aged <6 months,^4^ with high rates also observed in persons aged ≥65 years.^20^ We found that, in central China, influenza-associated hospitalization rates among persons ≥65 years were much lower than for young children, but are slightly higher than that the rates estimated in subtropical Bangladesh 12 and in the temperate USA.^20^ The highest influenza-associated hospitalization rate in our study was in infants aged 6–11 months and the second highest in those <6 months. This is different from that observed in the USA, where the highest rate was observed in infants<6 months, although that study did not provide specific data on children aged 6–11 months.^4^

To our knowledge, our study is one of the first to report hospitalization rates attributable to influenza by viral type/subtype. A few small cohort studies reported the proportion of children presenting at the emergency room in France^21^ and in USA,^22^ and the proportion of health care visits in the USA,^6^,^23^ caused by influenza B and A(H3N2). Influenza B-associated hospitalization rates in our study during 2011–2012 were more than twice the rates associated with A(H3N2). This finding suggests that illness caused by influenza B virus were at least as severe as A(H3N2) viral infections in one season, which is different than one study in which estimated excess hospitalization rates associated with influenza B were lower than for A(H3N2) using a time series modeling approach.^24^

Influenza viral strains associated with SARI in Jingzhou were generally similar to the predominant strains detected by the national influenza surveillance system in the region. In our study, similar to A(H3N2) viruses, influenza B viruses were associated with a disproportionate number of hospitalizations in children aged <5 years during the 2-year surveillance period, which is different from the perception that influenza B illness appears to be highest among older children and young adults. Multiple studies have reported that children with influenza B are older than those with influenza A.^25^–^28^ In two studies that described influenza illness among children and adults, the proportion of illnesses caused by influenza B relative to influenza A was highest for individuals aged 5–29 years 29 and 2–39 years,^30^ respectively.

There are a number of reasons why influenza-associated hospitalization rates may vary across time, countries, and climates. Direct comparisons may be limited by differences in case definitions, diagnostic methods, age stratification, study period, influenza activity and seasonality, influenza vaccine coverage, health care utilization, structure of the health care system, population age structure, and clinical criteria for hospital admission. Host immunity and virulence of influenza viral strains, such as pH1N1 virus or antigenically drifted strains, may also contribute to differences. Compared with other studies, we used a broad SARI case definition for enrollment. Compared to estimates of excess influenza-associated deaths using an indirect method approach – statistical models,^18^ few studies have used a direct method approach to estimate mortality attributable to laboratory-confirmed influenza. This may be because deaths caused by influenza may be unrecognized or attributed to co-morbidities or to secondary complications, particularly among the elderly.^31^,^32^ Our study population had a lower prevalence of known chronic medical conditions compared with that reported in the USA^4^–^7^ and may be partially explained by an overall lower prevalence of chronic diseases among the general Chinese population.^33^

Obtaining accurate data about hospitalizations associated with influenza is important for allocating resources for diagnosis, treatment, and the prevention of respiratory pathogens. Further studies are needed to elucidate how much variability in hospitalization rates is attributable to health-seeking behavior and physician practice and how much to differences in host and pathogen factors. One important next step will be identifying what pathogens are responsible for the 87% of SARI patients who tested negative for influenza viruses, including *Streptococcus pneumoniae* and *Haemophilus influenzae* type B, the leading bacterial causes of childhood pneumonia.^34^,^35^

The results in our study have important implications for influenza prevention in China. Seasonal influenza vaccination is not included in the national immunization program, and individuals must purchase it themselves. China CDC currently recommends annual influenza vaccination for persons with chronic illness, pregnant women, individuals aged <5 or ≥60 years old, health care workers, and close contacts of high-risk individuals,^36^ an estimated population of 570 million.^14^ China does not currently have the capacity to produce this much influenza vaccine.^14^ Many in China perceive that influenza does not contribute substantially to morbidity, but our study demonstrates that many persons are hospitalized annually for influenza, particularly children aged <5 years. Our results support China CDC's recommendations and strongly suggest that influenza vaccination should be targeted at young children if the goal is to reduce severe complications of influenza.

Our study is subject to several limitations. First, this study was only conducted for a 24-month period, and the influenza disease burden findings may not reflect hospitalization rates over many years. Second, we may have under-estimated influenza-associated hospitalizations, because we required that patients have an elevated temperature when screened within 24 hours after admission. A substantial population of hospitalized influenza patients do not have fever, particularly the elderly,^37^,^38^ and influenza viral infection can exacerbate underlying conditions resulting in hospitalization and death from non-respiratory illness, such as myocardial infarction and diabetic ketoacidosis.^39^–^42^ Diagnostic techniques, such as reverse transcription polymerase chain reaction (RT-PCR) and viral isolation, may yield false-negative results if samples are collected after viral shedding has ceased. This could also lead to under-estimation of influenza-associated hospitalizations. Third, SARI surveillance was not conducted at all hospitals, in the city. Fourth, the low median age and high estimated influenza-associated hospitalization rates for young children might be partially explained by the predominance of 2009 H1N1 and influenza B viruses during five of the seven periods of influenza activity under surveillance for this study. However, additional years of surveillance, especially during periods when H3N2 viruses are predominating, and health utilization survey data, are needed to assess the impact of influenza complications among elderly persons in Jingzhou and other areas of China. Finally, the findings from this study should not be generalized to all of China, where climate and other factors may cause variation in influenza burden.

Given the enormous population, geographic area, and diversity of climates, multiyear population-based surveillance with laboratory confirmation of viral and bacterial respiratory pathogens should be implemented in different geographical and climactic regions of China. Data from such studies will provide evidence to help prevent influenza and other respiratory infections in China. Continuing surveillance over several years and conducting similar studies in temperate and subtropical regions in China will help confirm our findings on influenza seasonality and may help improve our understanding of factors driving seasonality.

## Supporting Information

**Table S1.** Proportion of all hospitalized patients and influenza-associated hospitalized patients in four surveillance hospitals and in all health facilities that provide hospitalization services in Jingzhou and Shashi Districts, Jingzhou City, China, 2006–2010. **Table S2.** Comparison of characteristics between hospitalized SARI patients who had NP collected for influenza testing and those without NP collected during 24 months surveillance period (from April 5, 2010 to April 8, 2012) in Jingzhou, China. **Table S3.** Estimated hospitalization rates of SARI patients and rates attributable to influenza by age group in Jingzhou, China, April 5, 2010 to April 8, 2012.

## References

[1] United States Centers for Disease Control and Prevention http://www.cdc.Gov/flu/weekly/overview.htm.

[2] Barr IG, McCauley J, Cox N (2010). Epidemiological, antigenic and genetic characteristics of seasonal influenza A(H1N1), A(H3N2) and B influenza viruses: basis for the WHO recommendation on the composition of influenza vaccines for use in the 2009–2010 Northern Hemisphere season. Vaccine.

[3] WHO (2012). Interim Global Epidemiological Surveillance Standards for Influenza. http://www.who.int/influenza/resources/documents/INFSURVMANUAL.pdf.

[4] Dawood FS, Fiore A, Kamimoto L (2010). Burden of seasonal influenza hospitalization in children, United States, 2003 to 2008. J Pediatr.

[5] Grijalva CG, Weinberg GA, Bennett NM (2007). Estimating the undetected burden of influenza hospitalizations in children. Epidemiol Infect.

[6] Neuzil KM, Zhu Y, Griffin MR (2002). Burden of interpandemic influenza in children younger than 5 years: a 25-year prospective study. J Infect Dis.

[7] Poehling KA, Edwards KM, Weinberg GA (2006). The underrecognized burden of influenza in young children. N Engl J Med.

[8] Izurieta HS, Thompson WW, Kramarz P (2000). Influenza and the rates of hospitalization for respiratory disease among infants and young children. N Engl J Med.

[9] Olsen SJ, Laosiritaworn Y, Siasiriwattana S (2006). The incidence of pneumonia in rural Thailand. Int J Infect Dis.

[10] Simmerman JM, Chittaganpitch M, Levy J (2009). Incidence, seasonality and mortality associated with influenza pneumonia in Thailand: 2005–2008. PLoS ONE.

[11] Chiu SS, Chan KH, Chen H (2009). Virologically confirmed population-based burden of hospitalization caused by influenza A and B among children in Hong Kong. Clin Infect Dis.

[12] Eduardo AB, Alamgir ASM, Rahman M (2012). Incidence of influenza-like illness and severe acute respiratory infection during three influenza seasons in Bangladesh, 2008–2010. Bull World Health Organ.

[13] Borja-Aburto VH, Chowell G, Viboud C (2012). Epidemiological characterization of a fourth wave of pandemic A/H1N1 influenza in Mexico, winter 2011–2012: age shift and severity. Arch Med Res.

[14] Feng L, Mounts AW, Feng Y (2010). Seasonal influenza vaccine supply and target vaccinated population in China, 2004–2009. Vaccine.

[15] National Bureau of Statistics of China http://www.stats.gov.cn/tjsj/pcsj/rkpc/6rp/indexch.htm.

[16] China Weather http://www.weather.com.cn/cityintro/101200801.shtml.

[17] WHO (2011). Manual for the Laboratory Diagnosis and Virological Surveillance of Influenza. http://whqlibdoc.who.int/publications/2011/9789241548090_eng.pdf.

[18] Feng L, Shay DK, Jiang Y (2012). Influenza-associated mortality in temperate and subtropical Chinese cities, 2003–2008. Bull World Health Organ.

[19] Nair H, Brooks WA, Katz M (2011). Global burden of respiratory infections due to seasonal influenza in young children: a systematic review and meta-analysis. Lancet.

[20] Dao CN, Kamimoto L, Nowell M (2010). Adult hospitalizations for laboratory-positive influenza during the 2005–2006 through 2007–2008 seasons in the United States. J Infect Dis.

[21] Dominique P, Sylviane L, Daniele T (2003). Influenza burden in children newborn to eleven months of age in a pediatric emergency department during the peak of an influenza epidemic. Pediatr Infect Dis.

[22] Jeffrey MB, Krow A, Per G (2009). Development and validation of a risk score for predicting hospitalization in children with influenza virus infection. Pediatr Emerg Care.

[23] Hite LK, Glezen WP, Demmler GJ (2007). Medically attended pediatric influenza during the resurgence of the Victoria lineage of influenza B virus. Int J Infect Dis.

[24] Thompson WW, Shay DK, Weintraub E (2004). Influenza-associated hospitalizations in the United States. JAMA.

[25] Daley AJ, Nallusamy R, Isaacs D (2000). Comparison of influenza A and influenza B virus infection in hospitalized children. J Paediatr Child Health.

[26] Esposito S, Cantarutti L, Molteni CG (2011). Clinical manifestations and socioeconomic impact of influenza among healthy children in the community. J Infect.

[27] Peltola V, Ziegler T, Ruuskanen O (2003). Influenza A and B virus infections in children. Clin Infect Dis.

[28] Shen CF, Huang SC, Wang SM (2008). Decreased leukocytes and other characteristics of laboratory findings of influenza virus infections in children. J Microbiol Immunol Infect.

[29] Grant KA, Carville K, Fielding JE (2009). High proportion of influenza B characterises the 2008 influenza season in Victoria. Commun Dis Intell.

[30] Olson DR, Heffernan RT, Paladini M (2007). Monitoring the impact of influenza by age: emergency department fever and respiratory complaint surveillance in New York City. PLoS Med.

[31] Robertson L, Caley JP, Moore J (1958). Importance of Staphylococcus aureus in pneumonia in the 1957 epidemic of influenza A. Lancet.

[32] Eickhoff TC, Sherman IL, Serfling RE (1961). Observations on excess mortality associated with epidemic influenza. JAMA.

[33] WHO http://www.who.Int/healthinfo/global_burden_disease/GBD_report_2004update_full.pdf.

[34] O'Brien KL, Wolfson LJ, Watt JP (2009). Burden of disease caused by *Streptococcus pneumoniae* in children younger than 5 years: global estimates. Lancet.

[35] Watt JP, Wolfson LJ, O'Brien KL (2009). Burden of disease caused by *Haemophilus influenzae* type b in children younger than 5 years: global estimates. Lancet.

[36] Chinese Center for Disease Control and Prevention http://www.chinacdc.cn/n272442/n272530/n3479265/n3479308/40232.html.

[37] Falsey AR, Cunningham CK, Bark WH (1995). Respiratory syncytial virus and influenza A infection in the hospitalized elderly. J Infect Dis.

[38] Walsh EE, Cox C, Falsey AR (2002). Clinical features of influenza A virus infection in older hospitalized persons. J Am Geriatr Soc.

[39] Bainton D, Jones GR, Hole D (1978). Influenza and ischaemic heart disease-a possible trigger for acute myocardial infarction?. Int J Epidemiol.

[40] Fleming DM, Cross KW, Pannell RS (2005). Influenza and its relationship to circulatory disorders. Epidemiol Infect.

[41] Li CK, Choi BCK, Wong TW (2006). Influenza-related deaths and hospitalizations in Hong Kong: a subtropical area. Public Health.

[42] Troppan KT, Bozic M, Santner BI, Kessler HH (2010). Evaluation of four molecular assays for detection of pandemic influenza A (H1N1) 2009 virus in the routinediagnostic laboratory. J Clin Virol.

