# Supplementary material for: The substantial hospitalization burden of influenza in central China: surveillance for severe, acute respiratory infection, and influenza viruses, 2010–2012
Source: Influenza Other Respir Viruses. 2013 Nov 10;8(1):53–65. doi: 10.1111/irv.12205 (PMC4177798; doi:10.1111/irv.12205)
Supplement: Supplementary file 1 [file irv0008-0053-SD1.doc]

**Supplementary Table 1: Proportion of all hospitalized patients and influenza-associated hospitalized patients in 4 surveillance hospitals and in all health facilities that provide hospitalization services in Jingzhou and Shashi Districts, Jingzhou City, China, 2006-2010.**

| Year | Jingzhou Central Hospital | Jingzhou First People’s Hospital | Jingzhou Second People’s Hospital | Jingzhou Maternal and Children's Hospital | No. of hospitalized patients in all health facilities that provide hospitalization services (no. of health facilities)^a^ | Proportion |
| --- | --- | --- | --- | --- | --- | --- |
| All hospitalized patients ^b^ | | | | | | |
| 2006 | 30,806 | 32,260 | 14,274 | 3,669 | 110,892 (29) | 0.73 |
| 2007 | 32,464 | 34,357 | 16,180 | 9,579 | 127,245 (32) | 0.73 |
| 2008 | 36,920 | 43,220 | 18,277 | 10,100 | 166,919 (47) | 0.65 |
| 2009 | 41,675 | 49,273 | 21,506 | 10,921 | 193,086 (48) | 0.64 |
| 2010 | 44,612 | 53,950 | 23,913 | 11,341 | 214,508 (48) | 0.62 |
| Influenza-associated hospitalized patients ^c^ | | | | | | |
| 2006 | 2,235 | 1,322 | 463 | 3,669 | 10,819 (29) | 0.71 |
| <12 months^d^ | 609 | 335 | 0 | 2,104 | 3,710 | 0.82 |
| 12 - 23 months | 174 | 135 | 61 | 733 | 1,300 | 0.85 |
| 2 - 4 yrs | 509 | 381 | 140 | 633 | 2,470 | 0.67 |
| 5 - 9 yrs | 215 | 207 | 101 | 175 | 1,118 | 0.62 |
| 10 - 14 yrs | 80 | 82 | 43 | 23 | 356 | 0.64 |
| 15 - 49 yrs | 258 | 127 | 64 | 1 | 784 | 0.57 |
| 50 - 64 yrs | 146 | 26 | 28 | 0 | 453 | 0.44 |
| ≥65 yrs | 244 | 28 | 26 | 0 | 609 | 0.49 |
| Missing | 0 | 1 | 0 | 0 | 19 | 0.05 |
| 2007 | 2,643 | 1,295 | 474 | 4,387 | 12,598 (32) | 0.70 |
| < 12 months | 713 | 273 | 0 | 2,260 | 3,915 | 0.83 |
| 12 - 23 months | 204 | 151 | 50 | 978 | 1,612 | 0.86 |
| 2 - 4 yrs | 721 | 411 | 137 | 949 | 3,240 | 0.68 |
| 5 - 9 yrs | 203 | 208 | 85 | 165 | 1,170 | 0.56 |
| 10 - 14 yrs | 59 | 62 | 37 | 33 | 349 | 0.55 |
| 15 - 49 yrs | 255 | 114 | 70 | 2 | 844 | 0.52 |
| 50 - 64 yrs | 157 | 47 | 48 | 0 | 605 | 0.42 |
| ≥65 yrs | 331 | 28 | 47 | 0 | 813 | 0.50 |
| Missing | 0 | 1 | 0 | 0 | 50 | 0.02 |
| 2008 | 3,512 | 1,683 | 552 | 4,607 | 15,060 (47) | 0.69 |
| < 12 months | 990 | 327 | 0 | 2,300 | 4,449 | 0.81 |
| 12 - 23 months | 309 | 145 | 55 | 946 | 1,771 | 0.82 |
| 2 - 4 yrs | 1,001 | 532 | 155 | 1,109 | 4,094 | 0.68 |
| 5 - 9 yrs | 308 | 277 | 113 | 212 | 1,375 | 0.66 |
| 10 - 14 yrs | 71 | 70 | 37 | 33 | 371 | 0.57 |
| 15 - 49 yrs | 261 | 179 | 59 | 5 | 977 | 0.52 |
| 50 - 64 yrs | 175 | 82 | 46 | 0 | 785 | 0.39 |
| ≥65 yrs | 397 | 71 | 87 | 0 | 1,076 | 0.52 |
| Missing | 0 | 0 | 0 | 2 | 162 | 0.01 |

^a^. No. of health facilities provide hospitalization services varies from years due to increasing investments on hospital expending.

^b^. Median of all hospitalized patients in 4 surveillance hospitals overall health facilities: 0.65 (range: 0.62, 0.73)

^c^. Median (range) of all influenza-associated hospitalized patients in 4 surveillance hospitals overall health facilities: 0.70 (0.69-0.71): 0.82 (0.81-0.83) for patients aged <12 months, 0.85 (0.82-0.86) for patients aged <12-23 months, 0.68 (0.67-0.68) for patients aged 2-4 years, 0.62 (0.56-0.66) for patients aged 5-9 years, 0.57 (0.55-0.64) for patients aged 10-14 years, 0.52 (0.52-0.57) for patients aged 15-49 years, 0.42 (0.39-0.44) for patients aged 50-64 years, 0.50 (0.49-0.52) for patients aged >65 years.

^d^, Median (range) of all influenza-associated hospitalized patients in 4 surveillance hospitals for patients under 6 months was estimated from patients aged <12 months which is 0.82 (0.81-0.83).

**Supplementary table 2:** Comparison of characteristics between hospitalized SARI patients who had NP collected for influenza testing and those without NP collected during 24 months surveillance period (from April 5, 2010 to April 8, 2012) in Jingzhou, China

| **Characteristics^1^** | **Patients with NP collected (n=16,208)** | **Patients without NP collected (n=964)** | **p value** |
| --- | --- | --- | --- |
| Male sex | 9,292 (57) | 575 (60) | 0.157 |
| Age, median years (IQR) | 2.2 (1.0-4.3) | 2.2 (1.0-4.5) | 0.593 |
| Age group |  |  |  |
| <6 months | 1,255 (8) | 63 (7) |  |
| 6 - 12 months | 2,947 (18) | 174 (18) | 0.060 |
| 12 - 23 months | 3,381 (21) | 215 (22) |
| 2 - 4 yrs | 5,030 (31) | 288 (30) |
| 5 - 9 yrs | 1,600 (10) | 110 (11) |
| 10 - 14 yrs | 291 (2) | 28 (3) |
| 15 - 49 yrs | 588 (4) | 29 (3) |
| 50 - 64 yrs | 437 (3) | 17 (2) |
| ≥65 yrs | 679 (4) | 40 (4) |
| *Illness onset by month* | | |  |
| 2010 |  |  |  |
| April | 43 (0.3) | 4 (0.4) | 0.258 |
| May | 145 (0.9) | 18 (2) |
| June | 327 (2) | 24 (2) |
| July | 365 (2) | 29 (3) |
| August | 492 (3) | 47 (5) |
| September | 541 (3) | 36 (4) |
| October | 386 (2) | 31 (3) |
| November | 716 (4) | 48 (5) |
| December | 788 (5) | 48 (5) |
| 2011 |  |  |  |
| January | 673 (4) | 54 (6) | **< 0.001** |
| February | 546 (3) | 20 (2) |
| March | 838 (5) | 30 (3) |
| April | 816 (5) | 40 (4) |
| May | 754 (5) | 39 (4) |
| June | 753 (5) | 57 (6) |  |
| July | 779 (5) | 59 (6) |  |
| August | 693 (4) | 49 (5) |  |
| September | 755 (5) | 32 (3) |  |
| October | 886 (5) | 29 (3) |  |
| November | 847 (5) | 50 (5) |  |
| December | 1038 (6) | 74 (8) |  |
| 2012 |  |  |  |
| January | 837 (5) | 43 (4) |  |
| February | 867 (5) | 37 (4) | 0.337 |
| March | 1130 (7) | 61 (6) |  |
| April | 193 (1) | 5 (0.5) |  |
| Temperature (T, ℃) |  |  |  |
| T> 38.0 | 9,712 (60) | 567 (59) | 0.598 |
| Admitted to intensive care unit | 148 (0.9) | 6 (0.6) | 0.352 |
| Died during hospitalization | 59 (0.4) | 3 (0.3) | 1.000 |

^1^Data is presented as no. (%) of patients unless otherwise indicated. Denominators for testing of fewer patients than full group are indicated. Percentages may not total 100 because of rounding. IQR, interquartile range.

**Supplementary Table 3: Estimated hospitalization rates of SARI patients and rates attributable to influenza by age group in Jingzhou, China, April 5, 2010 to April 8, 2012.**

| Age group | Population size of residents | Year ^a^ | No. of hospitalized SARI patients among residents | Proportion of SARI patients with NP collection for influenza testing among residents | No. of patients confirmed with influenza among residents | Proportion of influenza-associated hospitalized patients in the 4 surveillance hospitals among all health facilities, median (range) | Estimates of hospitalization rates of SARI patients per 100,000, median (range) ^b^ | Estimates of hospitalization rates attributable to influenza per 100,000, median (range) ^c, d^ |
| --- | --- | --- | --- | --- | --- | --- | --- | --- |
| <6 months | 3,605 | 2010-2011 | 457 | 0.93 | 74 | 0.82 (0.81-0.83) | 15,460 (15,273-15,650) | 2,692 (2,659-2,725) |
|  |  | 2011-2012 | 764 | 0.97 | 67 |  | 25,845 (25,533-26,164) | 2,337 (2,308-2,365) |
| 6-11 months | 4,285 | 2010-2011 | 945 | 0.94 | 119 | 0.82 (0.81-0.83) | 26,895 (26,571-27,227) | 3,603 (3,560-3,647) |
|  |  | 2011-2012 | 1,954 | 0.95 | 127 |  | 55,611 (54,941-56,297) | 3,805 (3,759-3,852) |
| 12 - 23 months | 8,320 | 2010-2011 | 1,205 | 0.94 | 153 | 0.85 (0.82-0.86) | 17,039 (16,841-17,662) | 2,302 (2,275-2,386) |
|  |  | 2011-2012 | 2,170 | 0.95 | 163 |  | 30,684 (30,328-31,807) | 2,426 (2,398-2,515) |
| Subtotal < 2 yrs | 16,210 | 2010-2011 | 2,607 | 0.94 | 346 | 0.82 (0.81-0.83) | 19,613 (19,377-19,855) | 2,769 (2,736-2,803) |
|  |  | 2011-2012 | 4,888 | 0.95 | 357 |  | 36,773 (36,330-37,227) | 2,827 (2,793-2,862) |
| 2 - 4 yrs | 23,519 | 2010-2011 | 1,890 | 0.94 | 273 | 0.68 (0.67-0.68) | 11,818 (11,818-11,994) | 1,816 (1,816-1,843) |
|  |  | 2011-2012 | 3,246 | 0.95 | 370 |  | 20,296 (20,296-20,599) | 2,435 (2,435-2,472) |
| Subtotal < 5 yrs | 39,729 | 2010-2011 | 4,497 | 0.94 | 619 | 0.82 (0.81-0.83) | 13,804 (13,638-13,974) | 2,021 (1,997-2,046) |
|  |  | 2011-2012 | 8134 | 0.95 | 727 |  | 24,968 (24,667-25,276) | 2,349 (2,321-2,378) |
| 5 - 9 yrs | 37,645 | 2010-2011 | 581 | 0.94 | 86 | 0.62 (0.56-0.66) | 2,489 (2,338-2,756) | 392 (368-434) |
|  |  | 2011-2012 | 1,094 | 0.93 | 208 |  | 4,687 (4,403-5,189) | 958 (900-1061) |
| 10 - 14 yrs | 44,927 | 2010-2011 | 104 | 0.94 | 9 | 0.57 (0.55-0.64) | 406 (362-421) | 37 (33-39) |
|  |  | 2011-2012 | 208 | 0.89 | 40 |  | 812 (723-842) | 176 (156-182) |
| 15 - 49 yrs | 707,183 | 2010-2011 | 278 | 0.94 | 39 | 0.52 (0.52-0.57) | 76 (69-76) | 11 (10-11) |
|  |  | 2011-2012 | 307 | 0.96 | 51 |  | 83 (76-83) | 14 (13-14) |
| 50 - 64 yrs | 224,903 | 2010-2011 | 234 | 0.95 | 54 | 0.42 (0.39-0.44) | 248 (236-267) | 60 (57-65) |
|  |  | 2011-2012 | 208 | 0.98 | 23 |  | 220 (210-237) | 25 (24-27) |
| ≥65 yrs | 99,699 | 2010-2011 | 355 | 0.94 | 66 | 0.50 (0.49-0.52) | 712 (685-727) | 141 (135-144) |
|  |  | 2011-2012 | 347 | 0.95 | 42 |  | 696 (669-710) | 89 (85-90) |
| Total | 1,154,086 | 2010-2011 | 6,049 | 0.94 | 873 | 0.70 (0.69-0.71) | 749 (738-760) | 115 (113-117) |
|  |  | 2011-2012 | 10,298 | 0.95 | 1,091 |  | 1,275 (1,257-1,293) | 142 (140-144) |

^a^2010-2011: from April 5, 2010 to April 3, 2011; 2011-2012: from April 4, 2011 to April 8, 2012.

^b^Estimates of hospitalization rates of SARI patients = No. of SARI patients among residents/population size/proportion of influenza-associated hospitalized patients in 4 surveillance hospitals among all health facilities which provide hospitalization services. We excluded non-residents both from the numerator and denominator to estimated hospitalization rates.

^c^Estimates of hospitalization rates attributable to influenza = No. of patients confirmed with influenza among residents/population size/proportion of patients with NP collection for influenza testing among residents/proportion of influenza-associated hospitalized patients in 4 surveillance hospitals among all health facilities which provide hospitalization services. We excluded non-residents both from the numerator and denominator to estimated hospitalization rates.

^d^Using the proportion of hospitalized patients seen at the four surveillance hospitals, rather than only influenza-associated hospitalized patients, to adjust our hospitalization estimates, produced similar results (data not shown).
